# The RASSF1A Tumor Suppressor Binds the RasGAP DAB2IP and Modulates RAS Activation in Lung Cancer

**DOI:** 10.3390/cancers12123807

**Published:** 2020-12-17

**Authors:** Desmond R. Harrell Stewart, M. Lee Schmidt, Howard Donninger, Geoffrey J. Clark

**Affiliations:** 1 Department of Pharmacology & Toxicology, University of Louisville School of Medicine, Louisville, KY 40202, USA; desmond.stewart@louisville.edu (D.R.H.S.); lschmidt@usworldmeds.com (M.L.S.); 2 Department of Medicine, University of Louisville School of Medicine, Louisville, KY 40202, USA; howard.donninger@louisville.edu

**Keywords:** RASSF1A, DAB2IP, KRAS, lung cancer

## Abstract

**Simple Summary:**

The RASSF1A tumor suppressor can serve as a pro-apoptotic effector of the K-RAS oncoprotein. It is frequently inactivated epigenetically in lung cancer, and genetic inactivation of RASSF1A in transgenic mice enhances the ability of mutant K-RAS to promote tumorigenesis. Here we show that RASSF1A complexes with and stabilizes the protein DAB2IP. DAB2IP is a tumor suppressor itself and acts, in part, as a negative regulator (GAP) for RAS. Thus, loss of RASSF1A results in the reduced expression of DAB2IP, which promotes the activation of wild type RAS. Therefore, RASSF1A negative cells are likely to show enhanced RAS activity. This may be the first example of a RAS effector being able to back-regulate RAS activity.

**Abstract:**

Lung cancer is the leading cause of cancer-related death worldwide. Lung cancer is commonly driven by mutations in the RAS oncogenes, the most frequently activated oncogene family in human disease. RAS-induced tumorigenesis is inhibited by the tumor suppressor RASSF1A, which induces apoptosis in response to hyperactivation of RAS. RASSF1A expression is suppressed in cancer at high rates, primarily owing to promoter hypermethylation. Recent reports have shown that loss of RASSF1A expression uncouples RAS from apoptotic signaling in vivo, thereby enhancing tumor aggressiveness. Moreover, a concomitant upregulation of RAS mitogenic signaling upon RASSF1A loss has been observed, suggesting RASSF1A may directly regulate RAS activation. Here, we present the first mechanistic evidence for control of RAS activation by RASSF1A. We present a novel interaction between RASSF1A and the Ras GTPase Activating Protein (RasGAP) DAB2IP, an important negative regulator of RAS. Using shRNA-mediated knockdown and stable overexpression approaches, we demonstrate that RASSF1A upregulates DAB2IP protein levels in NSCLC cells. Suppression of RASSF1A and subsequent downregulation of DAB2IP enhances GTP loading onto RAS, thus increasing RAS mitogenic signaling in both mutant- and wildtype-RAS cells. Moreover, co-suppression of RASSF1A and DAB2IP significantly enhances in vitro and in vivo growth of wildtype-RAS cells. Tumors expressing wildtype RAS, therefore, may still suffer from hyperactive RAS signaling when RASSF1A is downregulated. This may render them susceptible to the targeted RAS inhibitors currently in development.

## 1. Introduction

There are three human isoforms of the RAS oncogene—*HRAS, KRAS,* and *NRAS*—that collectively represent the most frequently activated oncogene in human cancer [1]. RAS possesses a highly potent transforming power, capable of activating several classical mitogenic pathways such as RAF-MEK-ERK, Phosphatidylinositol-3 kinase (PI3K)-AKT, and RAL guanine nucleotide exchange factor (RALGEF)-RAL [2]. RAS is a small GTPase that functions as a molecular binary switch, cycling between ON and OFF states by binding and hydrolyzing GTP [3]. The RAS GTPase cycle is tightly regulated; activating GDP-GTP exchange and inactivating GTP hydrolysis are controlled by two families of regulatory proteins, guanine nucleotide exchange factors (GEFs) and GTPase activating proteins (GAPs), respectively [4].

In cancer, RAS is rendered constitutively active by two main mechanisms: activating mutations and disruption of its regulators. Hyperactivating point mutations occur at RAS loci in up to 30% of tumors across all tissue types [1,5]. Overexpression/activating mutations in GEFs, or more commonly in the receptor systems that control them, and suppression/mutation of GAPs have also been reported [6,7,8,9]. RAS mutations generally function by abolishing intrinsic GTPase activity or inhibiting GAP-mediated catalysis of GTP hydrolysis [4,10]. Loss of GAP activity through mutation or downregulation has a similar effect, locking RAS in the GTP-bound state [9]. Some mutations, such as the KRAS^G12C^ mutation, retain intrinsic GTPase activity, instead adopting a “fast-cycling” character [11]. Interestingly, KRAS^G12C^ is the predominant RAS mutation in human lung cancer [1].

In addition to stimulating multiple mitogenic pathways, RAS also possesses the paradoxical ability to induce apoptosis and senescence [12]. Many of these effects are mediated through the RAL guanine nucleotide dissociation stimulator (RALGDS)/AF6 RAS association domain family (RASSF) of effector proteins, the best characterized being RASSF1A [13]. The best characterized functions of RASSF1A are RAS-dependent activation of Hippo and Bcl-2 associated X protein (BAX) pro-apoptotic signaling programs [14,15,16]. Loss of RASSF1A expression, primarily through promoter hypermethylation, is a frequent event in cancer [17]. Experimental inactivation of RASSF1A in vitro and in vivo uncouples RAS from these pathways and leads to enhanced growth and tumorigenesis of RAS-driven cells [18,19,20,21].

Clinically, the most aggressive human lung tumors are those in which RAS—predominantly K-RAS—has been mutated and the *RASSF1A* promoter has been hypermethylated [22]. These tumors often confer the poorest prognosis [22]. We have previously modeled this phenotype in mice, where we showed that induced expression of an oncogenic *kras* mutant in *rassf1a^+/–^* mice increased both the frequency of tumor formation and tumor size compared to *rassf1a^+/+^* littermates [21]. However, when we compared the signaling pathways between the RASSF1A-wildtype and heterozygous tumors, in addition to observing a loss of HIPPO pathway activation, we also observed a striking upregulation of all three main RAS mitogenic pathways. It has previously been shown that RASSF1A can modulate the PI3K-AKT pathway [23,24]. RASSF1A is also part of a regulatory complex involving RAF [25,26]. However, these interactions are complex and poorly understood. As we observed activation of all three canonical RAS effectors—RAF, PI3K, and RALGDS—upon suppression of RASSF1A, we hypothesized that RASSF1A might be modulating RAS activation itself. 

Using a yeast two-hybrid system with RASSF1A as bait, we identified the protein Disabled homolog 2 interacting protein (DAB2IP) as a direct binding partner. DAB2IP is an important GAP for RAS that is frequently downregulated in human tumors [9]. DAB2IP is an important regulator of multiple cellular processes, including inflammation, angiogenesis, transformation, proliferation, and metastasis [27,28,29,30,31]. This led us to the hypothesis that RASSF1A regulates RAS activation status by modulating the activity of DAB2IP. 

Here, using two non-small cell lung carcinoma (NSCLC) cell lines, one harboring a fast-cycling *KRAS* mutation and one with wildtype *RAS*, we show that RASSF1A suppression downregulates DAB2IP. Moreover, re-expressing RASSF1A in a RASSF1A null cell line upregulates DAB2IP protein levels. Furthermore, suppression of RASSF1A and DAB2IP leads to a striking upregulation of RAS-GTP loading and mitogenic signaling. Dual suppression also has a significant effect on growth of the wild type RAS cell line. Thus, we identify a novel RAS regulatory mechanism that may explain the positive effects of RASSF1A downregulation on RAS mitogenic signaling. This may be the first example of a RAS effector back-regulating RAS activity via a GAP.

## 2. Results

### 2.1. RASSF1A Binds the RASGAP DAB2IP

Several groups have reported increases in the activity of RAS mitogenic signaling pathways after RASSF1A downregulation [23,25,26]. We observed a similar result in vivo [21]. As part of our investigations, we performed a yeast two-hybrid screen to identify novel binding partners for RASSF1A (Myriad Genetics, Salt Lake City, UT, USA). We identified the protein DAB2IP, which bound to the 37–120 amino acid fragment of RASSF1A. This region contains the Cysteine Rich Domain (CRD). 

**Figure 1:**
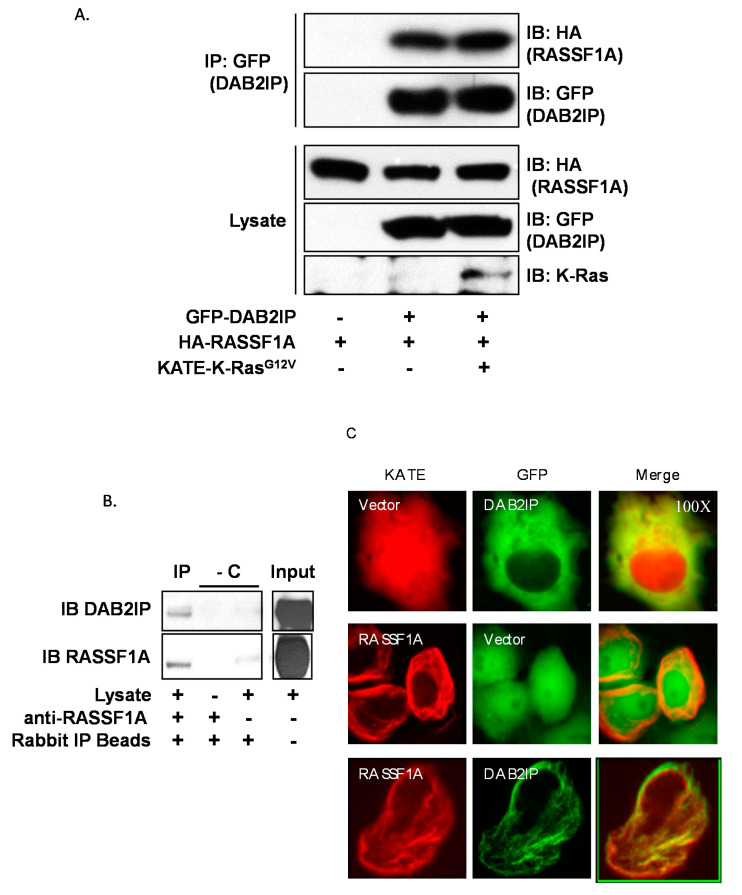
RASSF1A binds DAB2IP. IP = immunoprecipitation, IB = immunoblot, GFP = green fluorescent protein, HA = hemagglutinin, KATE = Katushka far-red fluorescent protein. (**A**) Co-immunoprecipitation of transiently overexpressed HA-RASSF1A with GFP-DAB2IP in HEK-293T cells in the presence or absence of activated K-RAS. Cells were plated and grown to 90% confluence, then transfected overnight. Cells were lysed in modified RIPA buffer. GFP was immunoprecipitated using antibody-conjugated beads and co-immunoprecipitation was detected via anti-HA Western blot. (**B**) Endogenous co-immunoprecipitation of DAB2IP with RASSF1A in un-transfected MCF10A breast epithelial cells. Two milligrams whole cell lysate prepared using modified RIPA buffer was incubated overnight with anti-RASSF1A antibody. Anti-RASSF1A was immunoprecipitated using anti-rabbit IP beads. Co-immunoprecipitation of DAB2IP was detected via anti-DAB2IP Western blot. Two IP reactions, one excluding the lysate and one excluding the anti-RASSF1A IP antibody, served as negative controls. -C = negative controls. (**C**) Fluorescence microscopy showing co-localization of transiently expressed GFP-DAB2IP with KATE-RASSF1A in COS-7 cells (100x). Cells were plated at low density on glass-bottom dishes and transfected overnight. Images were acquired on an inverted microscope under oil immersion.

To validate the two-hybrid result, we first performed overexpression followed by co-immunoprecipitation assays (Figure 1A). We anticipated that the interaction between RASSF1A and DAB2IP might be dependent on the activation state of RAS, so we performed immunoprecipitations in the presence and absence of a constitutively active K-RAS mutant. Interestingly, we found that RASSF1A readily co-immunoprecipitated with DAB2IP irrespective of the presence of the RAS mutant. To confirm the physiological relevance of the interaction, we sought to detect the endogenous interaction between RASSF1A and DAB2IP (Figure 1B). We used MCF-10A breast epithelial cells, a non-transformed epithelial line that expresses relatively high amounts of RASSF1A compared to cancer cell lines. Indeed, DAB2IP co-immunoprecipitated with RASSF1A in these cells, thus confirming that DAB2IP is a novel binding partner of RASSF1A. The stoichiometry appeared low, possibly indicating the interaction is dynamic and transient. Finally, we performed fluorescent microscopy studies in live cells. RASSF1A localizes prominently to microtubules [32,33,34]. When co-expressed, RASSF1A recruited DAB2IP from the cytosol to the microtubules (Figure 1C). Full length blots are provided in Figure S1.

### 2.2. RASSF1A Modulates DAB2IP Expression

**Figure 2:**
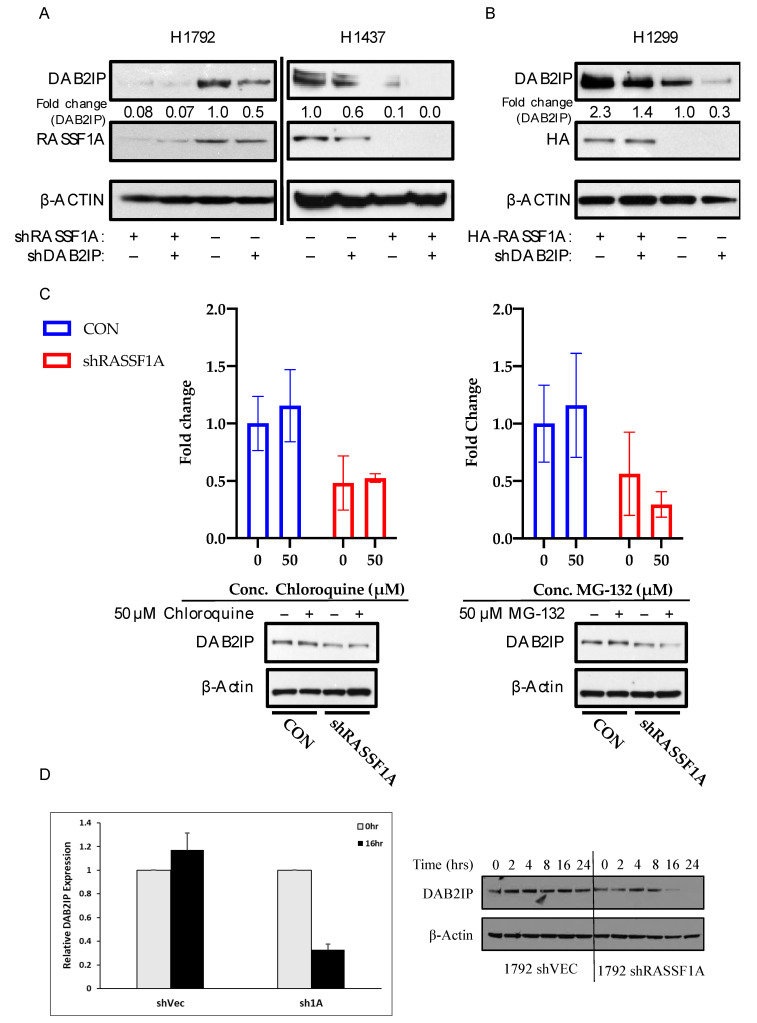
RASSF1A upregulates DAB2IP. (**A**) DAB2IP and RASSF1A expression in stably transfected NCI-H1792 and NCI-H1437 knockdown matched sets. Cells were grown to confluence and lysed in RIPA buffer. One hundred micrograms whole cell lysate was resolved per sample for Western blot analysis. (**B**) DAB2IP expression in NCI-H1299 HA-RASSF1A stably overexpressed system. Cells were grown to confluence and lysed in RIPA buffer. Twenty micrograms whole cell lysate was resolved per sample for Western blot analysis. (**C**) Chloroquine and MG-132 treatment in NCI- H1792 cells. Cells were treated with the above listed concentrations of chloroquine and MG-132, or an equivalent volume of DMSO for 24 or 4 h, respectively. Cells were lysed in RIPA buffer. Fifty micrograms whole cell lysate was resolved per sample. DAB2IP expression was determined by Western blot and quantified via densitometry. Reported as mean ± s.e., *n* = 2. (**D**) NCI-H1792 cells stably transfected with shRNA against RASSF1A or control vector were treated with cycloheximide at 25 μg/mL to inhibit protein synthesis and the relative stability of endogenous DAB2IP measured by Western blot over a time course. At time 16 h the results were quantified relative to time zero. Data are an average of two experiments. A representative blot is shown in the right panel.

RASSF1A and DAB2IP are frequently suppressed by promoter hypermethylation in lung cancer [22,35,36]. We hypothesized that they might act as a tumor suppressor complex and that dual inactivation might have a synergistic effect on the transformed phenotype. To test this hypothesis, we generated stable single and dual RASSF1A/DAB2IP knockdown cell lines using shRNAs. Empty carrier vectors served as controls, such that for each cell line used, we generated a passage-matched set of four sublines expressing the four possible combinations of control and knockdown vectors for RASSF1A and DAB2IP: pBRS-control and pGIPZ-control (CON); pBRS-control and pGIPZ-shDAB2IP (shDAB2IP); pBRS-shRASSF1A and pGIPZ-control (shRASSF1A); and finally, pBRS-shRASSF1A and pGIPZ-shDAB2IP (shD2+F1A). We used NCI-H1792 cells, which harbor a fast-cycling oncogenic KRAS mutation (G12C) [37], and NCI-H1437 cells, which contain only wild-type RAS [38,39]. While validating our matched sets of knockdown cells, we observed that shRNA-mediated knockdown of RASSF1A caused a striking reduction in the levels of DAB2IP protein. (Figure 2A). Knockdown of RASSF1A in NCI-H1792 cells was achieved using an shRNA described in [16], whereas the NCI-H1437 cells express a different shRNA purchased from Origene (described in [40]). Thus, this is unlikely to be a nonspecific effect of the shRNA. Moreover, stable overexpression of RASSF1A in NCI-H1299 cells (which have lost endogenous expression of RASSF1A [41]) markedly upregulated DAB2IP (Figure 2B). Thus, it appears that RASSF1A modulates DAB2IP protein levels. However, neither lysosomal nor proteasomal inhibition using chloroquine or MG-132, respectively, restored DAB2IP expression to control levels when RASSF1A was knocked down in NCI-H1792 cells (Figure 2C). Despite these results, examination of protein stability using cycloheximide suppression of translation showed that a loss of RASSF1A appeared to decrease DAB2IP protein stability (Figure 2D). This suggests that the effect is working, at least in part, at a protein stability level. Full length blots are provided in Figure S2.

### 2.3. RASSF1A May Act with DAB2IP to Regulate RAS Activation

**Figure 3:**
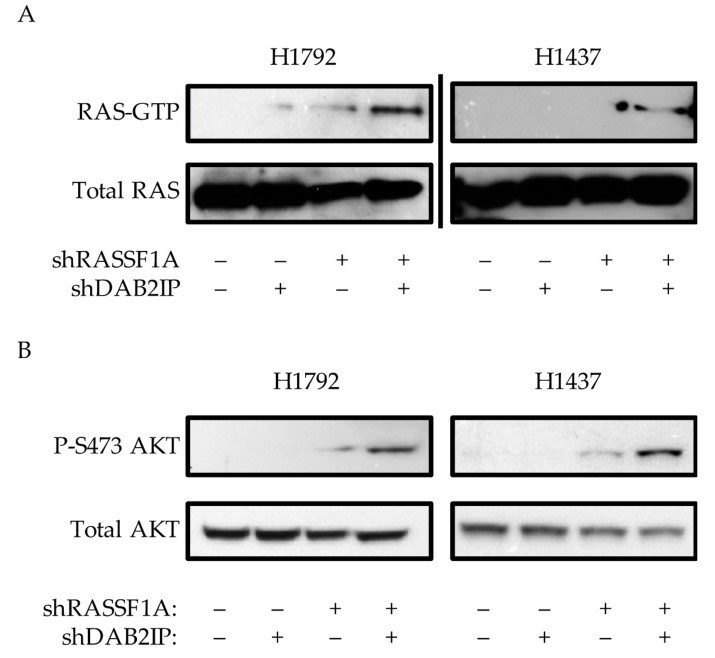
Effects of RASSF1A and DAB2IP suppression on RAS activity. (**A**) Active RAS pulldown assay on stably transfected NCI-H192 and NCI-H1437 knockdown cells, representative assays shown. (**B**) Western blot analysis of PI3K-AKT pathway activation. Representative assays shown of at least two independent experiments.

As DAB2IP is a GAP for RAS, we measured the effects of RASSF1A/DAB2IP suppression on RAS activation status. Although NCI-H1792 cells contain a mutant form of RAS, the mutant is a G12C mutation, which tends to act as a fast-cycling defect [37]. Therefore, even though mutant, it may still be sensitive to overall levels of GAP activity. We first measured the effects of RASSF1A/DAB2IP modulation on RAS-GTP levels. In both NCI-H1792 and NCI-H1437 cell systems we found that cells expressing shRNAs against both RASSF1A and DAB2IP exhibited a strong upregulation of RAS-GTP (Figure 3A), although it was strongest in the wild-type line. We then examined RAS signaling activity. Here we found that shRASSF1A-expressing cells exhibited increased activating phosphorylation at Ser437 of AKT in both NCI-H1792 and NCI-H1437 systems (Figure 3B). Co-suppression of RASSF1A and DAB2IP potentiated this effect. Full length blots are provided in Figure S3.

### 2.4. Dual Inhibition of RASSF1A and DAB2IP Has a Synergistic Effect on Growth and Transformation of a Wild-Type RAS Cell Line

**Figure 4:**
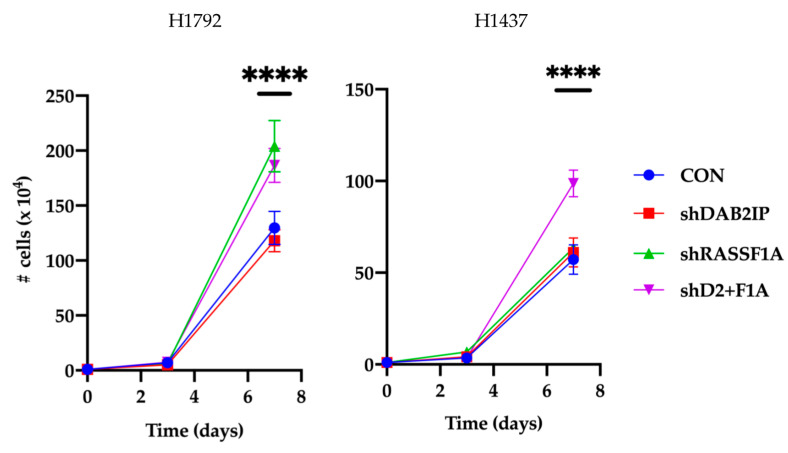
Effects of RASSF1A and DAB2IP suppression on in vitro 2D growth. Stably transfected NCI-H1792 and NCI-H1437 knockdown cells were plated at low density in 6-well plates. Triplicate wells were counted on a hemocytometer over the course of a week. Results are reported as mean ± s.e. of three independent experiments. ****, *p* < 0.0001, one-way ANOVA with Tukey’s test.

As we observed strongly upregulated RAS-GTP in both cell lines when RASSF1A and DAB2IP were inactivated together, we tested the cells to determine if this correlated with enhanced growth. In NCI-H1792 cells, we found that DAB2IP knockdown modestly increased 2D growth, whereas RASSF1A knockdown significantly increased growth compared to control cells (Figure 4). The addition of the DAB2IP shRNA to the RASSF1A knockdown cells had no additional significant effect on the growth. However, in the wild-type RAS NCI-H1437 cells, only co-suppression of RASSF1A and DAB2IP resulted in significantly increased 2D growth compared to control (Figure 4). 

**Figure 5:**
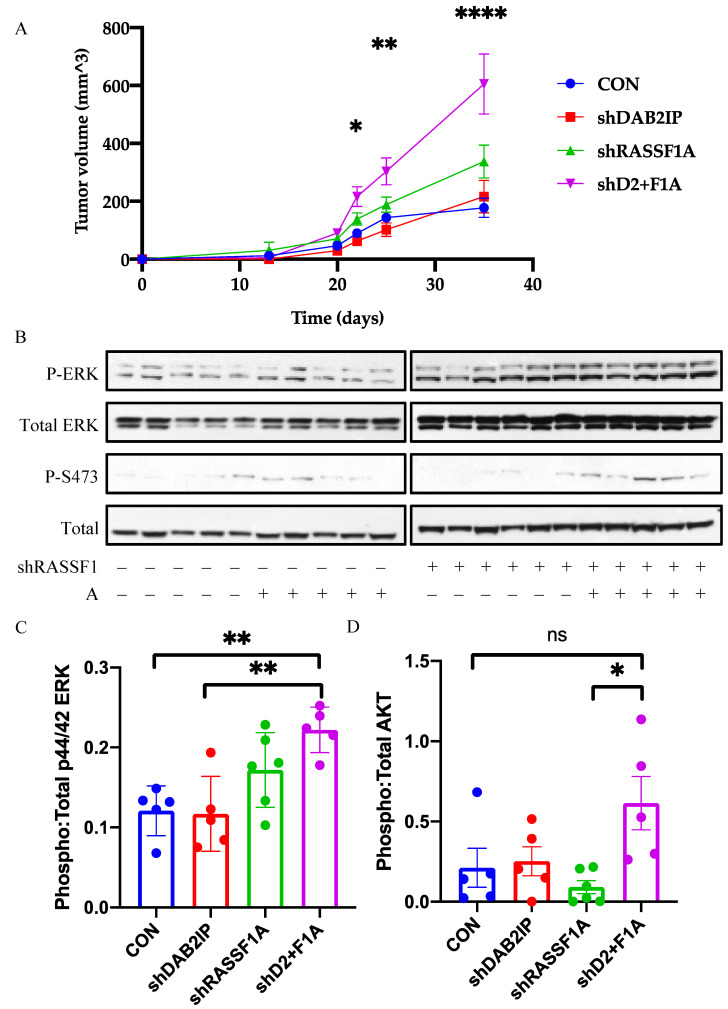
Effects of RASSF1A and DAB2IP suppression on in vivo growth and signaling. (**A**) NCI-H1437 xenograft growth curve. (**B**) Western blot analysis of RAS pathway activation in tumor lysates. (**C**,**D**) Densitometric analysis of MAPK pathway and PI3K-AKT pathway activation, respectively. Phospho and total protein images were obtained from the same nitrocellulose membrane. *n* = 5 or 6. * *p* < 0.05, ** *p* < 0.01, **** *p* < 0.0001, one-way ANOVA with Tukey’s test.

As the NCI-H1437 cells exhibited synergistic growth effects in 2D culture, we then tested them for the effects on tumor formation in immunocompromised mice. Suppression of DAB2IP or RASSF1A alone resulted in modest but insignificant increases in xenograft growth compared to control. However, co-suppression of RASSF1A and DAB2IP significantly enhanced tumor growth compared to control H1437 cells (Figure 5A). Analysis of RAS signaling in the tumors confirmed that the double shRNA transfected cells exhibited a significant upregulation of the RAS mitogenic pathway RAF-MEK-ERK, and a modest upregulation of PI3K-AKT (Figure 5B–D). Full length blots are provided in Figure S4.

## 3. Discussion

RAS oncoproteins may be the most important proteins in human cancer. They are deregulated by point mutation or loss/gain of function of their regulatory proteins in a majority of cases [42]. Upon activation, RAS activates multiple mitogenic pathways via its well characterized effectors RAF, PI3K, and RalGDS [43]. Paradoxically, excessive RAS stimulation can also induce apoptosis or senescence [12]. The mechanisms underlying these cell death pathways remain much less well characterized. The RASSF family of RAS effectors, particularly RASSF1A, have been shown to mediate many of these effects [13,44].

RASSF1A is suppressed in cancer at high rates by promoter hypermethylation. In fact, methylation of the *RASSF1A* promoter may be the most frequent tumor suppressor methylation event in cancer [17]. RASSF1A, like all RASSF proteins, lacks enzymatic activity, instead functioning as a scaffold that facilitates crosstalk between multiple, sometimes competing signaling pathways. Because of this, exactly how RASSF1A works is complex and remains poorly understood. It binds to K-RAS and can mediate K-RAS-induced apoptosis through activation of apoptotic signaling pathways such as HIPPO [14]. However, we have seen that suppression of RASSF1A not only uncouples RAS from the HIPPO pathway but also upregulates RAS mitogenic pathways [21,45]. Similar effects have been reported by other groups [23,25,26], but the mechanism underlying these effects is unclear. 

In this study, we sought to gain a more complete understanding of how loss of RASSF1A expression exacerbates RAS-mediated tumorigenesis. Having previously demonstrated that *rassf1a* haploinsufficiency could elevate RAS mitogenic activity, we searched for novel binding partners of RASSF1A that may confer regulation of RAS. We identified and confirmed a novel, direct protein–protein interaction between RASSF1A and a key regulator of RAS activity, the RASGAP DAB2IP. Like RASSF1A, DAB2IP is often downregulated in human tumors [9].

We speculated that RASSF1A and DAB2IP might form a tumor suppressor complex that acted in part by regulating RAS-GTP levels. This would explain the general upregulation of mitogenic RAS pathways when RASSF1A is suppressed. We chose to investigate the biological effect of this novel tumor suppressor complex using shRNA knockdown. This method of gene suppression more closely mimics the promoter methylation that occurs in tumors, where expression is reduced but rarely completely absent. The use of a RAS mutant cell line, which is generally resistant to GAP activity, to investigate the function of a GAP seems counterintuitive. However, use of the K-RAS^G12C^ mutant specifically is warranted here. First, unlike more common G12D or G12V mutations, K-RAS^G12C^ actively cycles between GTP- and GDP-bound states, and so may still be partially regulated by RASGAPs [37]. Second, the non-mutant forms of RAS remaining in the cell may be upregulated by GAP depletion, thereby supporting the effects of the mutant isoform [15]. Certainly, in our mouse model we observed increases in total RAS signaling over that due to mutant RAS when we downregulated RASSF1A [21]. Finally, this mutant, though relatively uncommon when considering all cancers, is the predominant mutation in lung cancer [1].

Our initial hypothesis was that RASSF1A might serve to couple DAB2IP to RAS in a feedback system. However, we saw no changes in the interaction between DAB2IP and RASSF1A in the presence of activated K-RAS. Instead, we observed a striking loss of endogenous DAB2IP protein expression when we downregulated RASSF1A in both cell lines. Further studies showed that overexpression of RASSF1A enhanced the expression levels of DAB2IP. This shows that RASSF1A may be acting on RAS by regulating the levels of its GAP, DAB2IP. Indeed, when we examined the knockdown sublines of NCI-H1437 and NCI-H1792 cells, we observed marked upregulation of RAS-GTP levels in cells knocked down for RASSF1A and DAB2IP. This was true even in the K-RAS^G12C^-containing NCI-H1792 cells. The results measuring the RAS-GTP levels were supported by Western analysis of the PI3K-AKT RAS mitogenic signaling pathway. The precise mechanism underlying the effects of RASSF1A on DAB2IP protein levels remains unclear. Inhibitors of the proteasome and lysosome had no effect on the process. However, general protein synthesis inhibition experiments suggested that DAB2IP protein was less stable in the absence of RASSF1A. Moreover, *DAB2IP* was not identified as a gene that is transcriptionally regulated by RASSF1A [46]. This suggests the regulation occurs at a protein level.

Analysis of the biological effects of single or dual knockdown of RASSF1A/DAB2IP showed that dual knockdown had the most dramatic effects on cell growth in the wild-type RAS cell line. In the mutant RAS cell line, however, while RASSF1A suppression enhanced growth, additional suppression of DAB2IP had no extra effect despite exhibiting elevated levels of RAS-GTP. Perhaps the enhanced levels of RAS-GTP due to RASSF1A inactivation alone were sufficient to maximally activate growth, and so a further increase in RAS-GTP had no additional effect in these cells. Conversely, this discrepancy in biology between the two cell lines may indicate that the observed effects on growth are not dependent on RAS signaling, or at least not completely. Both RASSF1A and DAB2IP are complex tumor suppressors that regulate multiple cellular processes, including growth, apoptosis, and genomic stability [45,47,48,49]. Further investigation is needed to determine how the effects of RASSF1A/DAB2IP on cell growth relate to their effect on RAS activation. 

As the wild-type RAS cell line exhibited the clearest cooperativity between RASSF1A and DAB2IP knockdown, we examined this cell line for the ability to form tumors in mice. The xenograft assays also showed a cooperative effect on tumor formation of RASSF1A and DAB2IP suppression. Analysis of signaling effects in lysates from the tumors confirmed the upregulation of RAS mitogenic signaling pathways. In addition to being a RASGAP, DAB2IP is a tumor suppressor with multiple RAS-independent activities [45]. It is possible that these activities also contribute to the enhanced transformation observed in the double knockdown cells. 

The cellular activities of RASSF1A are complex. First characterized as a pro-apoptotic RAS effector, RASSF1A has emerged in recent years as a potential regulator of RAS mitogenic activity. Our recent work has shed some light on exactly why RASSF1A loss is so permissive to transformation. By uncoupling RAS from a GAP, RASSF1A loss activates RAS. By uncoupling RAS from tumor suppressor pathways at the same time, it prevents the hyperactivated RAS from inducing cell death.

Recent work has raised the distinct possibility that the development of targeted inhibitors of RAS as therapeutic agents may be possible [11,50,51,52] These studies suggest that tumors without *ras* mutations, but with dual inactivation of RASSF1A and DAB2IP, may be sensitive to such agents.

## 4. Materials and Methods

### 4.1. Plasmids and shRNAs

RASSF1A and K-RAS expression constructs have been described previously [16,40]. GFP-DAB2IP was a gift from Karen Cichowski (Harvard, Boston, MA, USA). pBRS-shRASSF1A was generated by swapping the shRNA from pRS-RASSF1A 777 [53] into the pBRS-GFP vector (Origene, Rockville, MD, USA). shRASSF1A 777 localizes to exon 1α of the *RASSF1* gene, and thus suppresses expression of RASSF1A, D, E, F, G, and H while retaining expression of RASSF1B and C [19]. pGIPZ-shDAB2IP was a gift from Jer-Tsong Hsieh (UT Southwestern, Dallas, TX, USA) [54].

### 4.2. Tissue Culture and Cell Lines

HEK-293T and COS-7 cells were cultured in Dulbecco’s modified Eagle medium (DMEM) (Corning, Corning, NY, USA) with 10% fetal bovine serum (FBS) (Corning) and 1:100 dilution of penicillin/streptomycin (Corning). NCI-H1299, NCI-H1792, and NCI-H1437 cells were cultured in RPMI 1640 (Corning) with 10% FBS and 1:100 dilution of penicillin/streptomycin. MCF-10A cells were cultured in DMEM/Ham’s F12 50:50 Mix (Corning) supplemented with 10 μg/mL human recombinant insulin, 0.5 mg/mL hydrocortisone, 20 ng/mL epidermal growth factor (EGF), 5% horse serum (Life Technologies, Carlsbad, CA, USA) and 1:100 dilution of penicillin/streptomycin.

All stable cell lines were expanded and frozen as aliquots of an early-passage pooled population of cells. Stable NCI-H1437 transfectants were generated by transfecting 5 μg of DNA using Lipofectamine 3000 (Invitrogen, Carlsbad, CA, USA) according to the manufacturer’s protocol. shDAB2IP and pGIPZ control vector transfectants were selected in 1.5 μg/mL puromycin, followed by selection of shRASSF1A and pBRS-GFP control vector transfectants in 6 μg/mL blasticidin.

Knockdown of RASSF1A in NCI-H1792 cells has been described previously [16]. The shRNA expressed against *RASSF1* in these cells localizes to exon 1α and thus retains expression of RASSF1B and C. shDAB2IP and pGIPZ control vector transfectants were generated in NCI-H1792 cells by lentiviral infection of the DAB2IP shRNA followed by sorting of GFP-positive cells on a MoFlo XDP cell sorter (Beckman Coulter, Brea, CA, USA)

NCI-H1299 pZipHA-RASSF1A cells have been described previously [40]. NCI-H1299 pZipHA-RASSF1A/pGIPZ-shDAB2IP and pGIPZ control vector transfectants were generated using Lipofectamine 3000 according to the manufacturer’s protocol. Stable transfectants were selected in 1.5 μg/mL puromycin.

Transient transfections were performed using jetPRIME (Polyplus transfection, Illkirch, France) according to the manufacturer’s protocol. 

### 4.3. D Growth Assay

Each line was plated in nine wells of 6-well plates at 1 × 10^4^ cells per well. Triplicate wells were counted 24 h later as day 0, then again on days 3 and 7. 

### 4.4. Xenograft Growth Assay

Cells were plated at low density and harvested in log phase growth. Cells were resuspended in PBS (Phosphate Buffered Saline) at a concentration of 10 × 10^6^/mL, and 100 μL containing 1 × 10^6^ cells was injected subcutaneously into the left flank of an NRG mouse (Jackson Laboratory, Bar Harbor, ME, USA). Tumor length and width were measured using calipers, and volume was calculated using the formula V = (L × W^2^)/2. Six animals were used per cell line. 

### 4.5. Pulldown Assays and Western Blot Analysis

For co-immunoprecipitations, cells were lysed in modified RIPA buffer (150 mM NaCl, 50 mM Tris pH 7.5, 1% Tergitol type NP-40). GFP was immunoprecipitated using GFP-nAb agarose (Allele Biotechnology, San Diego, CA, USA). Endogenous RASSF1A was immunoprecipitated using TrueBlot anti-rabbit Ig agarose beads (Rockland Immunochemicals, Pottstown, PA, USA) and an in-house rabbit polyclonal RASSF1A antibody [21]. RAS-GTP pulldown assays were performed using the Active RAS Pulldown Kit (Cytoskeleton Inc., Denver, CO, USA) according to the manufacturer’s protocol. For signaling assays, tumor and cell lysates were prepared using RIPA buffer (MilliporeSigma, St. Louis, MO, USA). Proteins were visualized via Western blot using the Novex NuPage gel system (Invitrogen). Anti-HA was obtained from Sigma. Anti-GFP (B2) was obtained from Santa Cruz Biotechnology (Santa Cruz, CA, USA). Anti-DAB2IP was obtained from Aviva Systems Biology (San Diego, CA, USA). Anti-RASSF1A (3F3) was obtained from Abcam. Phospho-ERK, phospho-AKT, total ERK, and total AKT antibodies were obtained from Cell Signaling Technology (Danvers, MA, USA). Anti-Actin was obtained from Sigma. For phosphorylated proteins, the phospho antibody was probed first. Blocking of the membrane was then repeated following by probing with the total protein antibody. Densitometry was performed using ImageJ.

### 4.6. Fluorescence Microscopy

GFP and RFP proteins were visualized in live cells in complete growth medium using an IX50 inverted system microscope (Olympus, Tokyo, Japan). Images were acquired using an attached SPOT camera (Diagnostic Instruments Inc., Sterling Heights, MI, USA) and merged using ImageJ.

## 5. Conclusions

The RASSF1A tumor suppressor antagonizes Ras-mediated transformation by inducing apoptosis in response to Ras hyperactivation. The frequent loss of RASSF1A expression in tumors thus permits unrestrained mitogenic signaling by Ras mutants. It now appears that loss of RASSF1A expression may itself constitute a hyperactive Ras state. By modulating protein levels of the RasGAP DAB2IP, RASSF1A is able to regulate the activation state of Ras. Therefore, many “wild-type Ras” tumors may indeed be driven in part by hyperactivated Ras. 

## Supplementary Materials

The following are available online at https://www.mdpi.com/2072-6694/12/12/3807/s1, Figure S1: Original Western blots image of Figure 1; Figure S2: Original Western blots image of Figure 2; Figure S3: Original Western blots image of Figure 3; Figure S4: Original Western blots image of Figure 5.
