# Supplementary figures and images for: The RASSF1A Tumor Suppressor Binds the RasGAP DAB2IP and Modulates RAS Activation in Lung Cancer
Source: Cancers (Basel). 2020 Dec 17;12(12):3807. doi: 10.3390/cancers12123807 (PMC7766191; doi:10.3390/cancers12123807)

Figure S1

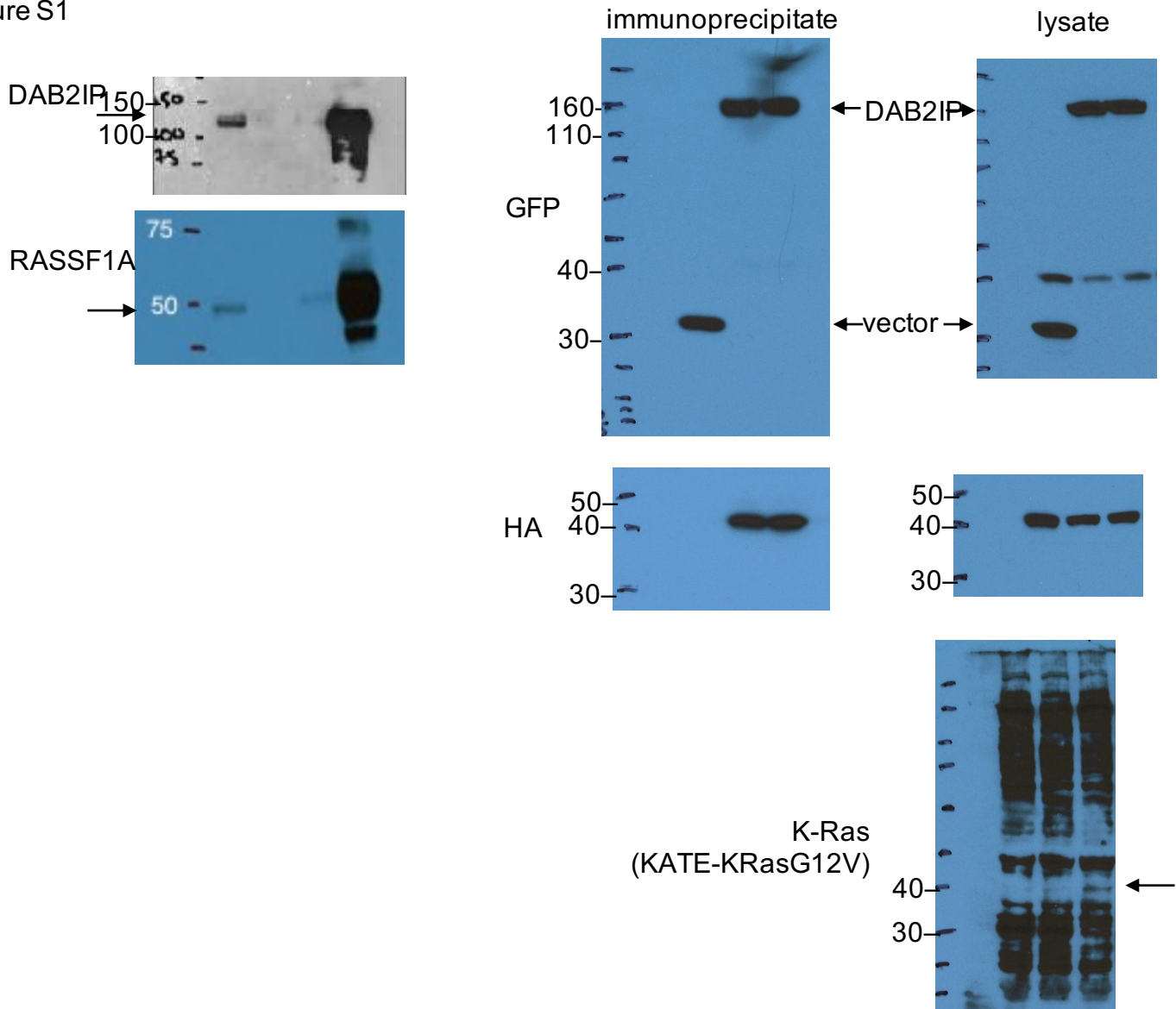

Figure S2

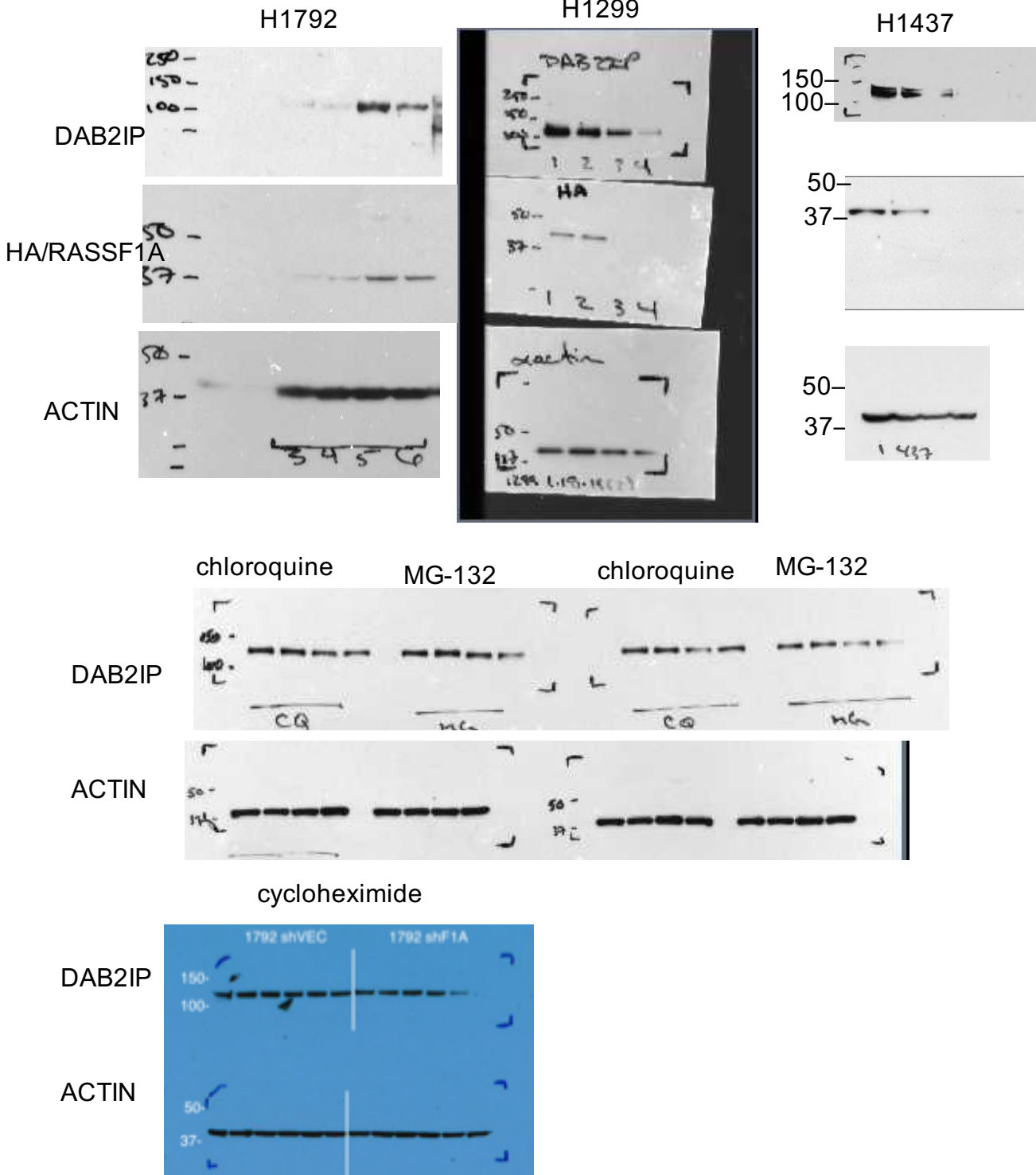

Figure S3

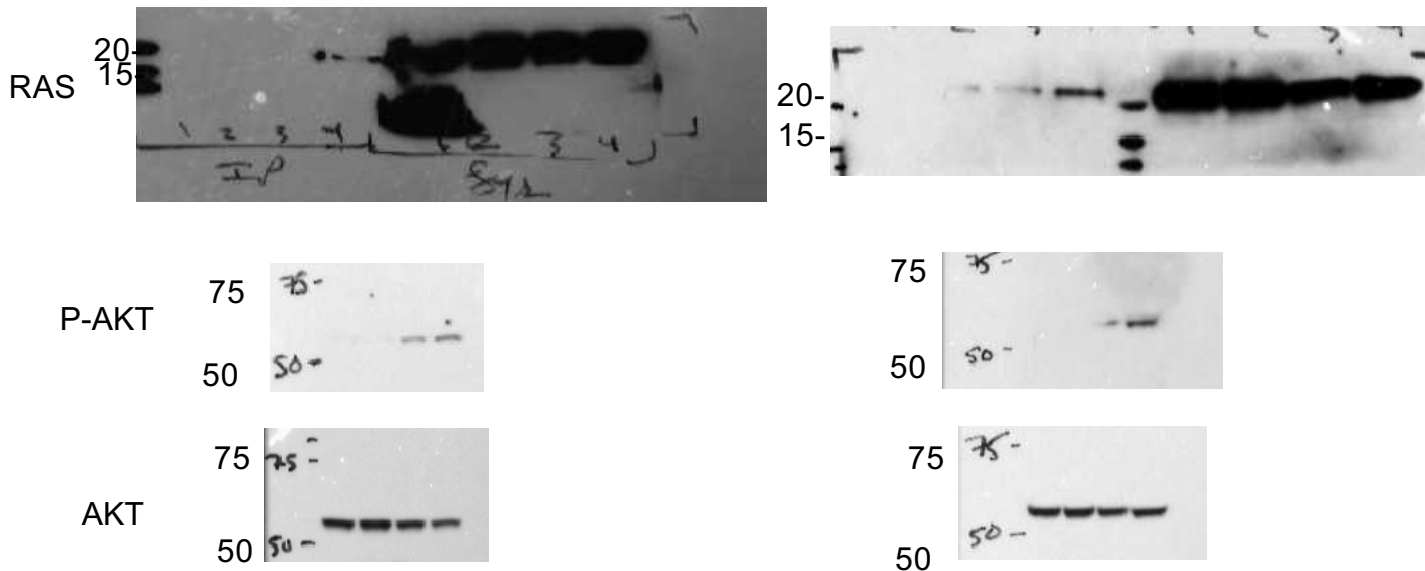

Figure S4

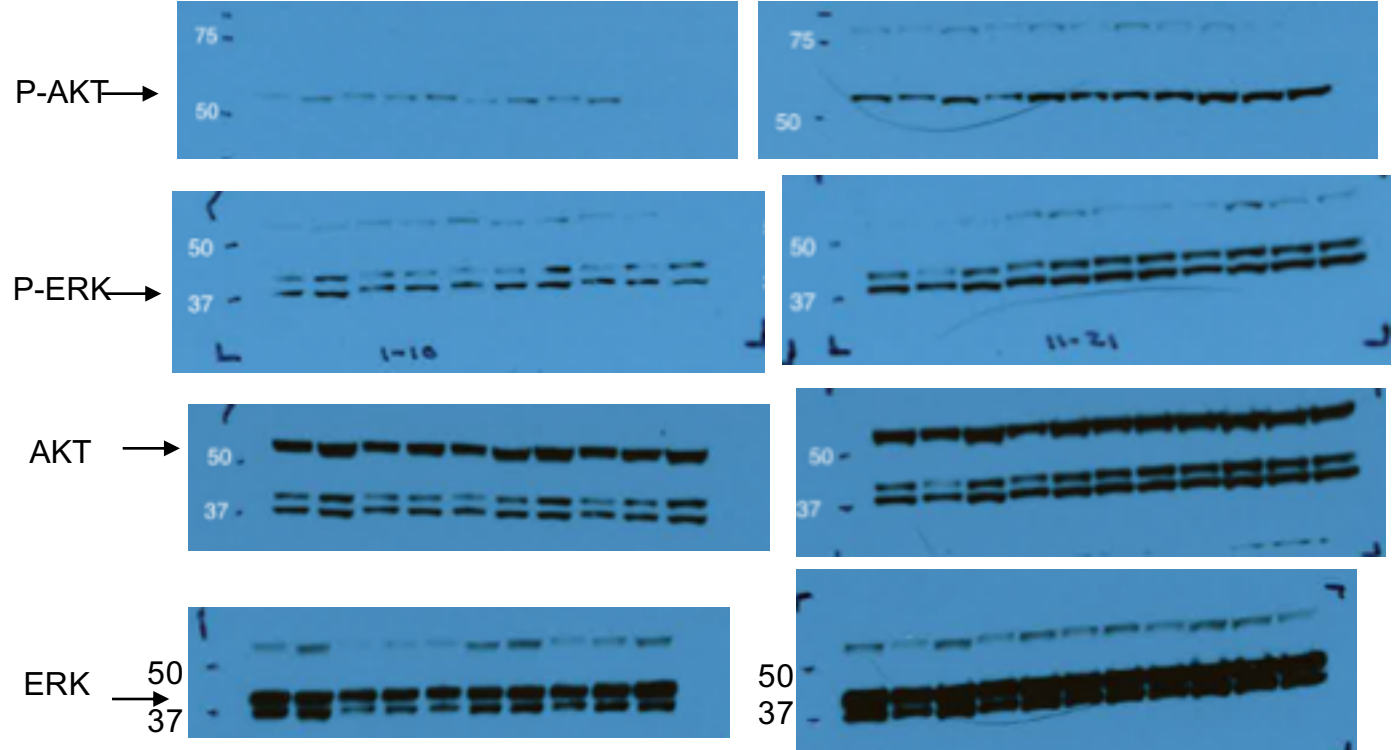

Supplement: Supplementary file 1 [file cancers-12-03807-s001.pdf]
